# Locked in Lucid Rhythms: A Case of Rhythmic Movement Disorder in Rapid Eye Movement (REM) Sleep

**DOI:** 10.7759/cureus.110405

**Published:** 2026-06-07

**Authors:** Muhammad Farhan, Rafeek Kandy, Bayena Alblooshi

**Affiliations:** 1 Psychiatry, Cleveland Clinic Abu Dhabi, Abu Dhabi, ARE; 2 Respiratory and Allergy Institute, Cleveland Clinic Abu Dhabi, Abu Dhabi, ARE; 3 Academics, Cleveland Clinic Abu Dhabi, Abu Dhabi, ARE

**Keywords:** antidepressants, hypnopompic hallucinations, narcolepsy, polysomnography, rem sleep, sleep paralysis, sleep-related rhythmic movement disorder

## Abstract

Sleep-related rhythmic movement disorder (SRRMD) is uncommon in adults. It can closely mimic nocturnal epilepsy and rapid eye movement (REM) sleep behavior disorder (RBD), creating significant diagnostic challenges. We report the case of a man in his mid-20s presenting with chief complaints of nightly head movements during sleep occurring multiple times each night. He intentionally shook his head to terminate these episodes, a volitional strategy he had developed to escape lucid dreams. Video-electroencephalogram (EEG) monitoring over two nights excluded epilepsy. Polysomnography (PSG) confirmed stereotyped lateral head rolling exclusively during REM sleep at approximately 1 Hz, without any loss of REM sleep atonia, thereby excluding RBD. Comorbid mild obstructive sleep apnea (apnoea-hypopnoea index (AHI) 8.7 events/hour) was also identified.

Nasal septoplasty partially improved symptoms. He reported excessive daytime sleepiness, with an initial Epworth Sleepiness Scale (ESS) score of 22. Due to the presence of excessive daytime sleepiness, two Multiple Sleep Latency Tests (MSLTs) were performed at different time points to evaluate for a primary hypersomnia disorder. However, both studies were conducted under suboptimal conditions. The first was performed in the presence of untreated mild sleep apnea, and the second while the patient was receiving medications known to suppress REM sleep. Neither study demonstrated sleep-onset REM periods, and mean sleep latencies of 7.2 and 12 minutes did not meet the diagnostic criteria for narcolepsy. Nevertheless, narcolepsy could not be definitively excluded because of the suboptimal testing conditions. The patient did not report symptoms suggestive of cataplexy. He experienced rare episodes of isolated sleep paralysis and a single episode of hypnopompic hallucination. The patient denied experiencing sleep attacks. He managed daytime sleepiness by scheduling daytime naps, which could last up to one to two hours. Sequential pharmacotherapy with amitriptyline, followed by nortriptyline, and subsequently the addition of venlafaxine and bupropion, achieved complete resolution of rhythmic movements and excessive daytime sleepiness.

We identified no previous reports describing a patient with successful management of rhythmic head movements during REM sleep using sequential tricyclic antidepressant (TCA), selective serotonin reuptake inhibitor (SSRI), and serotonin-norepinephrine reuptake inhibitor (SNRI) therapy for this condition. At the six-month follow-up, the patient's ESS score improved from 22 to 3.

## Introduction

Sleep-related rhythmic movement disorder (SRRMD) consists of stereotyped, repetitive movements during sleep, typically observed in childhood but occasionally persisting or emerging in adults [1]. According to the International Classification of Sleep Disorders, Third Edition Text Revision (ICSD-3-TR) [2], SRRMD is diagnosed when repetitive, stereotyped, and rhythmic motor behaviors involving large muscle groups occur predominantly during sleep and result in significant interference with normal sleep, daytime functional impairment, or self-inflicted bodily injury [2]. In adults, particularly when movements are associated with rapid eye movement (REM) sleep and accompanied by sleep paralysis, hypnopompic hallucinations, and lucid dream awareness, SRRMD can mimic nocturnal epilepsy, narcolepsy, or REM sleep behavior disorder (RBD), potentially prompting unnecessary investigations or treatments [1,2,3,4].

While most adult SRRMD cases represent persistence from childhood-onset, we report a case of adult-onset, REM-related SRRMD presenting with infrequent sleep paralysis and hypnopompic hallucinations, initially evaluated for epilepsy and narcolepsy, but ultimately managed as REM-related SRRMD. This case underscores the value of full-montage video-polysomnography (PSG), careful Multiple Sleep Latency Test (MSLT) interpretation, and targeted therapy that modulates REM sleep. To our knowledge, this is the first reported case of SRRMD in which a tricyclic antidepressant (TCA), a selective serotonin reuptake inhibitor (SSRI), and a serotonin-norepinephrine reuptake inhibitor (SNRI) were used with significant clinical benefit.

Written informed consent was obtained from the patient for the publication of this report.

## Case presentation

A man in his mid-20s, who was initially evaluated at an outside facility for disturbing nocturnal events, was referred to the neurology clinic at our centre for further assessment of possible epilepsy. He described the episodes as follows: "I feel like I am stuck in a dream, unable to move my body except for my head, which I shake to wake myself up." These episodes occurred almost nightly and during daytime naps. He explained, "If I fall asleep again too quickly, the same thing happens over and over. The only way to break the cycle is to sit up for a few minutes before trying to sleep again." He denied tongue biting, incontinence, or confusion upon awakening and consistently retained a clear recall of the events. There was no childhood history of similar symptoms or any family history of epilepsy or sleep disorders.

His medical history included psoriasis since childhood, psoriatic arthritis managed with biologic therapy, irritable bowel syndrome (IBS), generalized anxiety disorder, and asthma. He did not consume alcohol or illicit drugs and reported occasional vaping. Socially, he was employed while also pursuing further education. Review of systems was otherwise unremarkable, and both physical and neurological examinations were normal. A prior brain MRI from the outside facility was also unremarkable. Given the unusual nocturnal behaviors, video-electroencephalogram (EEG) monitoring was performed over two nights to evaluate for possible epilepsy. The study captured no epileptiform discharges. During a monitored nap, he experienced a frightened awakening without associated EEG changes or abnormal movements on video. Notably, in some episodes, he perceived that he was moving his head, although the video recording showed that he was not.

The patient was subsequently referred to the sleep clinic for further evaluation. He described persistent excessive daytime sleepiness with an Epworth Sleepiness Scale (ESS) score of 22, infrequent sleep paralysis, and rare hypnopompic hallucinations. He sometimes saw or heard family members upon awakening, only to realize they were not present. He also reported feeling sleepy after laughter but denied true cataplexy. There was no history of restless legs or substance use. Physical examination revealed a BMI of 22.8, a STOP-BANG score of 3, and a Mallampati classification of IV.

External PSG with next-day MSLT [7], performed before presentation at our centre, revealed mild obstructive sleep apnea (OSA) with an apnoea-hypopnoea index (AHI) of 8.7 events/hour and intermittent rhythmic head-rolling movements limited to REM sleep. Sleep architecture showed 26.4% REM sleep and 26.3% N3 sleep with REM latency of 69 minutes. The head movements were characterized as lateral head rolling during supine or lateral postures, clustered primarily in the first REM period, and terminating with brief arousals. The MSLT demonstrated a mean sleep latency of 7.2 minutes with no sleep-onset REM periods (SOREMPs).

**Figure 1:**
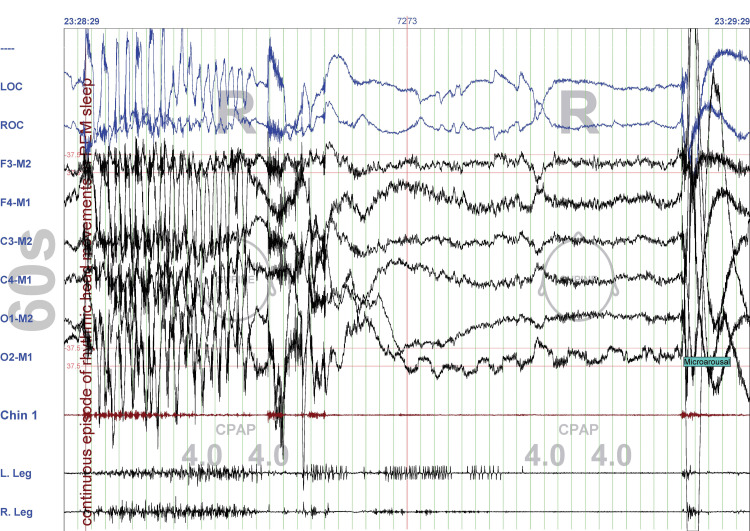
REM sleep epoch demonstrating rhythmic lateral head rolling One-minute video-polysomnographic epoch during REM sleep demonstrating continuous rhythmic movement artifact at approximately 1 Hz in the chin EMG channel and across EEG leads, corresponding to lateral head rolling confirmed on synchronized video REM: rapid eye movement; EMG: electromyogram; EEG: electroencephalogram

Upon presentation at our centre, continuous positive airway pressure (CPAP) titration was performed with optimal pressure determined at 8 cmH_2_O. The titration study also documented rhythmic head movements (lateral head rolling) during REM sleep at a frequency of approximately 1 Hz, with the longest episode lasting approximately 20 seconds (Figure 1). CPAP therapy was subsequently initiated, but adherence was suboptimal. The patient later underwent nasal septoplasty, which subjectively improved nasal breathing and reduced some nocturnal symptoms. Head movements during daytime naps persisted.

The patient continued to experience nocturnal head movements and fragmented sleep. Amitriptyline, 25 mg at bedtime, was initiated in an attempt to consolidate his nighttime sleep and to address IBS and headache symptoms. Within several weeks, the patient reported sleeping eight hours consecutively, with cessation of nocturnal head movements. He also noted improvement in his headache and irritable bowel symptoms, stating, "I finally started sleeping through the night, and the head movements stopped. My headaches and stomach problems also got better."

A repeat MSLT [7] was performed at our centre while the patient was on antidepressant therapy and had suboptimal CPAP adherence. The mean sleep latency was approximately 12 minutes, again with no SOREMPs. A protocol deviation was noted because the patient could not discontinue antidepressants due to the risk of symptom rebound, and CPAP adherence remained suboptimal. Over the following two to three months on amitriptyline, he experienced progressive weight gain. He was transitioned to nortriptyline, which was titrated to 50 mg at bedtime. This regimen maintained control of nocturnal symptoms. Despite these improvements in nighttime symptoms, he continued to experience head movements during daytime naps.

Fluoxetine 20 mg was added to his regimen to control head movements during daytime naps. Within several weeks, these movements substantially decreased. Unfortunately, he developed delayed ejaculation and additional weight gain. Fluoxetine was discontinued and replaced with venlafaxine 75 mg daily. Importantly, excessive daytime sleepiness improved substantially on nortriptyline and venlafaxine (ESS score 6) before the introduction of bupropion. Bupropion was then initiated and titrated from 150 mg to 300 mg daily specifically to counteract the sexual side effects of venlafaxine.

Over time, the patient's medication regimen was optimized to include nortriptyline 50 mg at bedtime, venlafaxine 75 mg daily, and bupropion 300 mg daily. This final medication regimen provided excellent control of both nocturnal and daytime symptoms, with complete resolution of rhythmic movements, sleep paralysis, and hypnopompic hallucinations. Throughout his treatment course, he declined stimulant therapy and hypocretin testing and received ongoing counseling on sleep hygiene and safety measures, including avoiding driving when sleepy. At last follow-up, he reflected, "I feel like I finally have control over my sleep again." At the six-month follow-up, his ESS score had improved from 22 to 3.

## Discussion

This case illustrates the diagnostic complexity when REM-linked rhythmic movements coexist with dissociated REM features, including sleep paralysis, hypnopompic hallucinations, and lucid awareness. This presentation created substantial diagnostic uncertainty across multiple sleep disorders. The patient's description of being "stuck in a dream" with intentional head shaking combined with objective rhythmic movements during REM sleep required systematic evaluation to distinguish among nocturnal epilepsy, RBD, narcolepsy, recurrent isolated sleep paralysis (RISP), and adult-onset SRRMD. The case was further complicated by comorbid mild OSA and protocol deviations during the second diagnostic MSLT testing that limited definitive characterization of the hypersomnolence. This complexity necessitated a comprehensive differential diagnosis approach, careful integration of clinical phenomenology with objective findings, and recognition of therapeutic overlap across potential diagnoses.

Nocturnal epilepsy was excluded based on normal video-EEG monitoring over two nights, absence of epileptiform activity, lack of seizure stigmata (tongue biting, incontinence, postictal confusion), and preserved event recall. Sleep-related epilepsy was also ruled out, given the clear REM association of movements rather than non-rapid eye movement (NREM) sleep onset.

**Figure 2:**
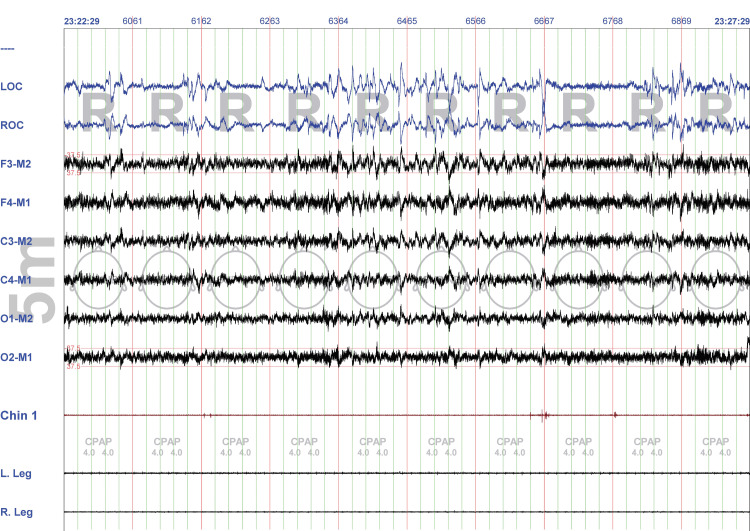
REM sleep epoch demonstrating low chin EMG tone without RSWA Five-minute video-polysomnographic epoch during REM sleep demonstrating low chin EMG tone without evidence of RSWA. Across all three polysomnographic studies, no RSWA was identified, arguing against REM sleep behavior disorder REM: rapid eye movement; EMG: electromyogram; RSWA: REM sleep without atonia

A diagnosis of RBD requires complex motor behaviors during REM sleep with documented REM sleep without atonia (RSWA) per ICSD-3-TR criteria [2]. Our patient exhibited only simple, stereotyped head-rolling movements without aggressive or goal-directed actions, sustained no injuries, and demonstrated no PSG evidence of RSWA in any of the PSGs (Figure 2). These features argue against RBD [8]. The movement artifact observed in the chin EMG channel during rhythmic movement epochs was confirmed on synchronized video as lateral head rolling rather than true RSWA. Outside these movement epochs, chin EMG tone remained consistently low throughout REM sleep across all three PSG studies, with no tonic or phasic EMG excess meeting RSWA criteria. Furthermore, antidepressants are well documented to precipitate or worsen RBD in susceptible individuals [9]; yet this patient improved consistently and progressively with escalating antidepressant doses, making RBD an unlikely diagnosis.

Narcolepsy diagnosis remained uncertain because the first MSLT was performed after noting mild sleep apnea on the baseline PSG, and the second MSLT was performed on REM-suppressing medications and with suboptimal CPAP adherence, both of which limit interpretation of the test per ICSD-3-TR and current American Academy of Sleep Medicine (AASM) MSLT guidelines [2,7]. The external MSLT (mean sleep latency 7.2 min, no SOREMPs, only four of five naps) indicated marked sleepiness but did not meet diagnostic criteria for narcolepsy type 1 or 2 [2]. Narcolepsy type 1 requires documented cataplexy with MSLT findings or low cerebrospinal fluid (CSF) hypocretin-1 (≤110 pg/mL); narcolepsy type 2 requires MSLT mean sleep latency ≤8 minutes with ≥2 SOREMPs and absence of cataplexy.

While sleep paralysis and hypnopompic hallucinations occur in 33% to 80% of narcolepsy patients, these dissociated REM phenomena also occur in other conditions and in individuals without narcolepsy [2]. CSF hypocretin measurement was declined by the patient. Importantly, excessive daytime sleepiness improved substantially during treatment with nortriptyline and venlafaxine (ESS score 6), before the introduction of bupropion. Bupropion was added specifically to counteract the sexual side effects of venlafaxine and was not prescribed to treat sleepiness. At the six-month follow-up on the complete regimen, the ESS score improved further to 3, consistent with antidepressant-mediated REM suppression rather than a stimulant effect.

RISP was considered, given sleep paralysis and hypnopompic hallucinations. RISP is defined as a transient inability to move or speak during sleep-wake transitions in the absence of other clinical features of narcolepsy [2]. While isolated episodes have a 15-40% lifetime prevalence, recurrent episodes with significant distress are less common [2]. However, key distinctions argued against RISP as the primary diagnosis, as the patient’s cardinal complaint was rhythmic head movements (used intentionally to terminate the episode), movements were objectively documented on PSG throughout REM periods (not just at awakening), and RISP does not present with visible motor phenomena on PSG.

Other sleep-related movement disorders were systematically excluded. Periodic limb movement disorder involves leg movements during NREM sleep with frequency >15 per hour in adults [2], rather than head movements during REM sleep. Sleep-related bruxism involves the jaw muscles with tooth grinding sounds. Propriospinal myoclonus occurs at sleep-wake transitions rather than during established REM sleep. Psychogenic movement disorders are absent during confirmed sleep on PSG. Adult REM-Related SRRMD best fit the phenotype based on multiple diagnostic features: stereotyped lateral head rolling at approximately 1 Hz (within the ICSD-3-TR diagnostic range of 0.5-2.0 Hz), occurrence exclusively during REM sleep, absence of RSWA on PSG, simple rather than complex motor patterns, preserved lucid awareness, and complete resolution with REM-suppressing antidepressants. While SRRMD typically occurs during NREM sleep or sleep-wake transitions, REM-predominant SRRMD occurs more frequently in adults than children [1,2].

OSA contributes to parasomnias through sleep fragmentation and arousal instability. OSA-induced arousals, particularly during REM sleep, can trigger or amplify motor phenomena through respiratory-related arousal mechanisms. Septoplasty likely reduced upper airway resistance and arousal burden, partially improving symptoms. However, complete resolution required REM-modulating pharmacotherapy, suggesting OSA was a modifier rather than the sole cause. This aligns with reports that OSA treatment produces variable improvement in adult SRRMD depending on residual arousal burden and CPAP adherence [1,10].

The therapeutic success with sequential REM-modulating medications (TCAs, SSRIs, SNRIs) supports REM-based pathophysiology. These agents suppress REM sleep through anticholinergic, serotonergic, and noradrenergic mechanisms, reducing REM percentage and increasing REM latency [11]. Tricyclic antidepressants enhance monoaminergic neurotransmission and exert anticholinergic effects, while SSRIs and SNRIs enhance serotonergic transmission from the dorsal raphe nucleus and noradrenergic transmission from the locus coeruleus, both of which are silent during REM sleep in normal physiology.

Importantly, REM-suppressing antidepressants are used therapeutically across multiple REM-related disorders, including narcolepsy (for cataplexy and dissociated REM phenomena), RISP, and REM-predominant SRRMD [12]. This therapeutic overlap means that treatment response alone cannot distinguish among these diagnoses. Diagnosis must be based on objective PSG findings, clinical phenomenology, and the systematic exclusion of alternatives. In this case, empiric treatment was reasonable given the patient's significant symptom distress and the adequate exclusion of epilepsy. The clinical response was consistent with this approach.

## Conclusions

This case expands the clinical spectrum of adult SRRMD by documenting adult-onset disease in the absence of a childhood history, the unique use of rhythmic movements as an intentional strategy to terminate a dissociated REM episode, and successful treatment with a combination of nortriptyline, venlafaxine, and bupropion (primarily to counter sexual side effects from an SSRI). In adults, REM-related SRRMD can mimic epilepsy, narcolepsy, and RBD. The co-occurrence of sleep paralysis and hypnopompic hallucinations further complicated the diagnostic evaluation, requiring the systematic exclusion of narcolepsy and other dissociated REM phenomena. A diagnostic approach combining video-EEG, video-PSG, and MSLT enabled targeted treatment despite diagnostic uncertainty. This case highlights the importance of multidisciplinary management, comprehensive differential diagnosis, recognition of therapeutic overlap across sleep disorders, and individualized treatment planning. Clinicians should maintain a high index of suspicion for this rare but treatable condition when evaluating adults with nocturnal motor phenomena and dissociated REM features.
